# Involvement of Ubiquitin-Conjugating Enzyme (E2 Gene Family) in Ripening Process and Response to Cold and Heat Stress of *Vitis vinifera*

**DOI:** 10.1038/s41598-017-13513-x

**Published:** 2017-10-16

**Authors:** Yingying Gao, Yi Wang, Haiping Xin, Shaohua Li, Zhenchang Liang

**Affiliations:** 1 Key Laboratory of Plant Germplasm Enhancement and Specialty Agriculture, Wuhan Botanical Garden, Chinese Academy of Sciences, Wuhan, 430074 PR China; 2 Beijing Key Laboratory of Grape Science and Enology and Key Laboratory of Plant Resource, Institute of Botany, Chinese Academy of Sciences, Beijing, 100093 PR China; 3 University of Chinese Academy of Sciences, Beijing, 100049 PR China; 4 Sino-Africa Joint Research Center, Chinese Academy of Sciences, Wuhan, 430074 PR China

## Abstract

Ubiquitin-conjugating (UBC) E2 enzyme plays crucial roles in plant growth and development. Limited information can describe the function of UBC enzyme E2 in grapes. A total of 43 UBC enzyme E2 genes with conserved UBC domain were identified in grapes. These genes were divided into five groups based on phylogenetic tree with tomatoes. Sequence analyses indicated that *VvUBCs* in the same group possessed similar gene structures and conserved motifs. Gene distribution in chromosomes was uneven, and gene duplication existed in 36 *VvUBCs*. Transcriptome and qRT-PCR analysis indicated that most *VvUBCs* are involved in ripening and post-harvest stage, and feature functional roles in grape organs. According to the transcriptome and qRT-PCR results, seven and six *VvUBCs* in grape responded to cold and heat stress, respectively, whereas no remarkable *VvUBCs* change was noted under salt or water-deficit stress. This study provides new insights to physiological and developmental roles of these enzymes and regulation mechanism of E2 genes in grapes.

## Introduction

Ubiquitination is an important type of post-translational modification of proteins among all eukaryotes. This important process regulates a wide range of biological processes^1^, including intracellular translocation of proteins, chromosomal organization, DNA repair, cell cycle control, and apoptosis^2–4^.

Ubiquitin covalently binds with target proteins, causing a series of enzyme catalytic effects. This process requires coordination of three types of enzymes, namely, ubiquitin-activating enzyme (E1), ubiquitin-conjugating (UBC) enzyme (E2), and ubiquitin-ligase enzyme (E3)^5^. Ubiquitin is activated in an ATP-dependent manner linked with E1; E2 accepts ubiquitin from E1, passes it to active-site cysteine, and then transfers ubiquitin to a targeted protein aided by E3^5^. Additional ubiquitin can be further ligated to initial ubiquitin molecule through sequential ubiquitination cycles, ultimately forming a poly- ubiquitin chain; finally, targeted proteins are modified^5^. Then, substrates can be degraded to generate other biological effects. E2 plays a crucial role in ubiquitination and is responsible for attachment of ubiquitin to targeted proteins^5^. E2 protein contains a conserved catalytic domain, called the UBC domain, spanning 140–200 amino acids in length. Various studies indicated that UBC domain mediates the interaction between E2 and E3^6–10^. A special interaction occurs between UBC domain in E2 and RING domain in E3^11^.

E2 genes exist as a multi-gene family and are involved in many plant physiological activities. A total of 14, 50, 41, 39, and 75 E2 genes were identified in *Saccharomyces cerevisiae* ^12^, humans^13^, *Arabidopsis* ^14^, rice^15^, and maize^16^, respectively. A number of E2 genes are involved in environmental stresses. For example, *VrUBC1* of mung bean responded to osmotic^17^ stress, and E2 genes in soybean and peanut reacted to drought and salt stress in transgenic *Arabidopsis* ^18–20^. Recently, researchers discovered that fruit-ripening regulator (RIN) can directly bind to the promoter of E2 genes in tomato, pigmentation of fruit was altered at orange ripening by silencing of E2 genes^21^. E2 genes are also involved in plant disease resistance throng positive plant immune regulation^22,23^. Some researchers observed association of E2 genes with cryogenic autolysis in *Volvariella volvacea* ^24^. *GhUNC1/2* is involved in auxin-associated effects and is related to degradation of target proteins, delaying senescence in cotton^25^.

Grapes (*Vitis vinifera*) are one of the most important fruit species in the world. Genome sequence of this fruit was released in 2007, it provides foundation for ongoing studies at the genome level^26^. At present, limited information can describe the role of E2 enzyme in grapes. For example, 45 E2 genes family members were identified in 8× coverage assembly of *Vitis vinifera* PN40024 genome^27^. E2-21 is down-regulated at veraison stage in Cabernet Sauvignon (*Vitis vinifera*)^28^. Until now, no systematical analysis has been performed on E2 genes family to identify their expression during grapevine development and response during abiotic stress.

The following are objectives of the present study: to identify and clarify members of E2 genes family from the 12× coverage assembly grapevine genome, to characterize their expression pattern during grapevine development and berry ripening, and to explore their functions in abiotic stress. Understanding functions of E2 enzymes bears significance in analyzing regulation mechanism of enzymes in grapevines.

## Results

### Identification of *Vitis vinifera* UBC enzyme E2 proteins

In this study, 43 unique UBC enzyme members were identified using *Hidden Markov Model* (HMM) and BLAST search methods (Table 1). All these genes contained the UBC domain. A phylogenetic tree was constructed, with 43 VvUBC members in grapes and 52 SlUBC members in tomatoes. VvUBCs showed the relationship between grapes and tomatoes on the phylogenetic tree.

**Table 1:** Information of *Vitis vinifera* ubiquitin-conjugating enzymes E2 gene family identified in this study.

Gene locus ID	Gene symbol	Protein length(aa)	Group	Chr	Start	End	NCBI Accession	Additional features
GSVIVT01026953001	*VvUBC1a*	184	V	15	19076770	19083025	CBI40397.3	C-terminal extension
GSVIVT01019387001	*VvUBC1b*	183	V	2	236398	241211	CBI34362.3	C-terminal extension
GSVIVT01024005001	*VvUBC3*	1098	IV	3	1786700	1794856	CBI37856.3	N&C-terminal extension
GSVIVT01011359001	*VvUBC4*	119	III	14	28935655	28941655	CBI22169.3	—
GSVIVT01005206001	*VvUBC5a*	944	IV	Un	19190912	19196143	CBI23966.3	N&C-terminal extension
GSVIVT01005576001	*VvUBC5b*	271	IV	Un	40650232	40651612	CBI25934.3	C-terminal extension
GSVIVT01009784001	*VvUBC7*	161	I	18	11241271	11245493	CBI19762.3	—
GSVIVT01009655001	*VvUBC8*	197	IV	18	10233988	10235820	CBI19650.3	—
GSVIVT01008045001	*VvUBC9*	152	III	17	6349780	6356685	CBI15257.3	—
GSVIVT01022074001	*VvUBC10*	162	III	7	16377832	16385165	CBI21382.3	—
GSVIVT01024027001	*VvUBC11*	148	II	3	1661190	1662457	CBI37874.3	—
GSVIVT01025431001	*VvUBC12*	528	II	6	847758	858488	CBI16509.3	N-terminal extension
GSVIVT01022467001	*VvUBC15*	119	III	8	3378128	3385686	CBI39063.3	—
GSVIVT01016663001	*VvUBC16*	305	I	9	206133	216225	CBI35791.3	N&C-terminal extension
GSVIVT01016569001	*VvUBC17*	168	IV	13	2698670	2704723	CBI31693.3	—
GSVIVT01018860001	*VvUBC19*	161	I	4	19153005	19160933	CBI17438.3	—
GSVIVT01027045001	*VvUBC20*	148	II	15	18385250	18393574	CBI40471.3	—
GSVIVT01034196001	*VvUBC21*	168	IV	8	14510755	14517473	CBI30575.3	—
GSVIVT01024998001	*VvUBC22*	176	IV	6	5391170	5399972	CBI16161.3	—
GSVIVT01019018001	*VvUBC23*	183	IV	4	17682215	17686563	CBI17566.3	—
GSVIVT01024546001	*VvUBC24*	148	II	6	8975439	8978143	CBI15805.3	—
GSVIVT01011671001	*VvUBC25a*	159	I	1	5344900	5350070	CBI26841.3	—
GSVIVT01033925001	*VvUBC25b*	146	I	8	16675006	16681479	CBI30364.3	—
GSVIVT01036063001	*VvUBC25c*	146	I	6	21209023	21218935	CBI28272.3	—
GSVIVT01016300001	*VvUBC25d*	190	I	13	5369865	5375890	CBI31480.3	N-terminal extension
GSVIVT01020056001	*VvUBC26*	191	III	1	11008741	11013679	CBI32005.3	N-terminal extension
GSVIVT01008615001	*VvUBC27*	150	I	17	394286	397861	CBI15730.3	C-terminal extension
GSVIVT01009448001	*VvUBC29*	160	III	18	8465118	8469528	CBI19485.3	—
GSVIVT01015392001	*VvUBC30*	188	I	11	3245946	3247764	CBI28077.3	—
GSVIVT01020551001	*VvUBC31*	472	IV	12	4458311	4466998	CBI21855.3	N&C-terminal extension
GSVIVT01020701001	*VvUBC32*	153	I	12	3005962	3011245	CBI21980.3	—
GSVIVT01014758001	*VvUBC33*	148	II	19	9349179	9350972	CBI39803.3	—
GSVIVT01019484001	*VvUBC34*	148	II	2	1020201	1030408	CBI34447.3	—
GSVIVT01025872001	*VvUBC36*	148	II	8	10943294	10948994	CBI32888.3	—
GSVIVT01014343001	*VvUBC38*	497	IV	19	2820615	2830331	CBI20306.3	N&C-terminal extension
GSVIVT01014215001	*VvUBC39*	153	I	19	1528687	1547441	CBI20200.3	—
GSVIVT01028729001	*VvUBC40*	148	II	16	19346927	19354509	CBI22557.3	—
GSVIVT01025833001	*VvUBC44*	311	I	8	11398555	11422512	CBI32855.3	N&C-terminal extension
GSVIVT01031547001	*VvUBC45*	177	I	6	17270831	17272824	CBI17191.3	—
GSVIVT01035654001	*VvUBC46*	157	I	4	3045208	3049485	CBI20878.3	—
GSVIVT01007794001	*VvUBC47*	184	V	17	8952004	8971372	CBI15064.3	C-terminal extension
GSVIVT01031919001	*VvUBC51*	183	IV	3	5404938	5409770	CBI32552.3	—
GSVIVT01035008001	*VvUBC52*	297	I	5	854146	857902	CBI22894.3	C-terminal extension

–represents no additional features.

### Phylogenetic analysis of VvUBC family

**Figure 1:**
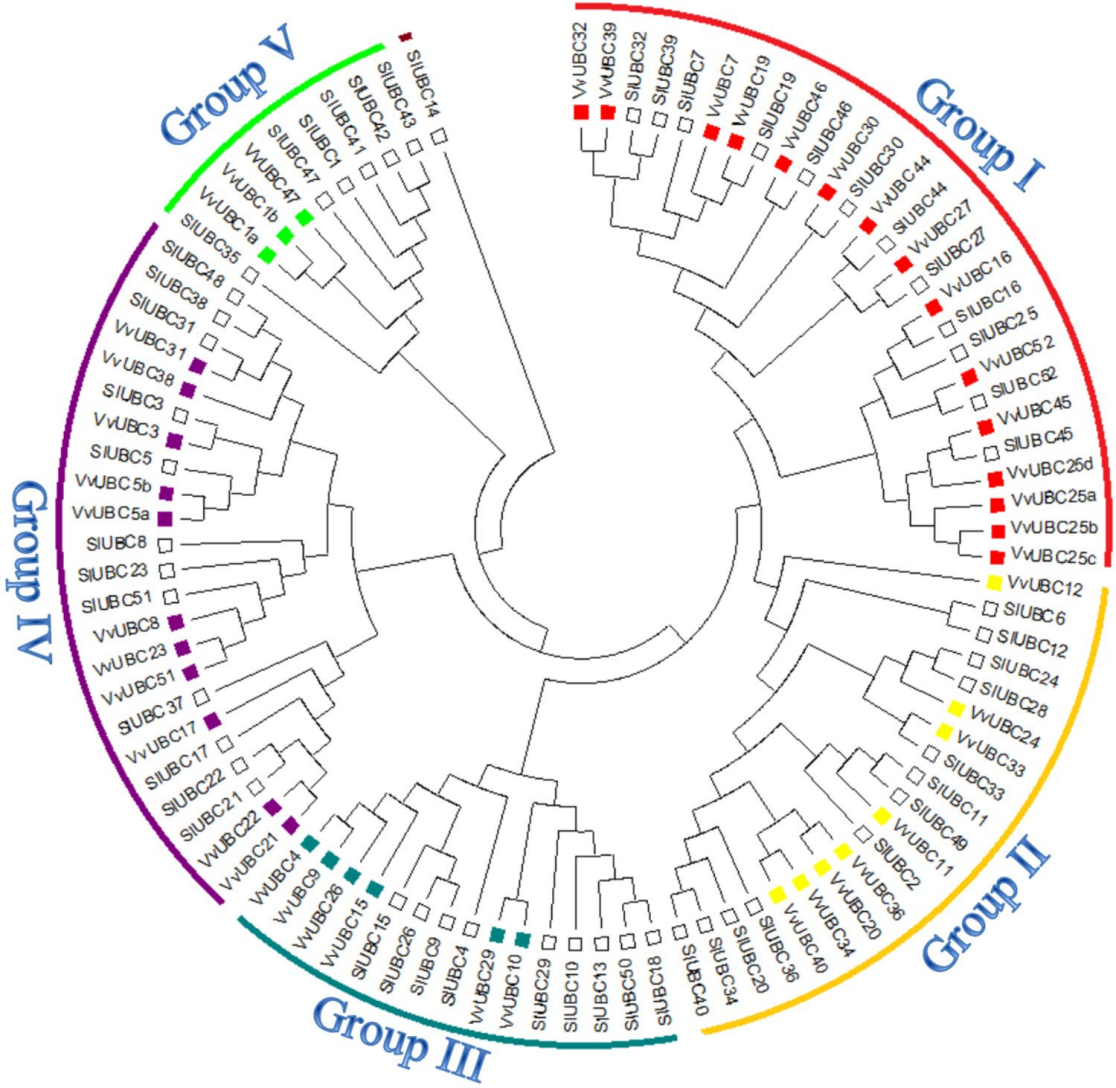
Phylogenetic tree of ubiquitin-conjugating enzyme E2 members among *Vitis vinifera* (43) and *Solanaceae lycopersicon* (52).

Phylogenetic analysis showed that 43 VvUBC members can be classified into five groups (Fig. 1 and Table 1). Groups I to V (Table 1) contained 15, 8, 6, 11, and 3 members, respectively. Compared with grapes, SlUBC members in tomatoes were classified into six groups. Group I, II, and IV each included 12 members. Group III and V contained 9 and 6 members, respectively. SlUBC14 existed in Group VI alone (Fig. 1).

### Conserved domain analysis

**Figure 2:**
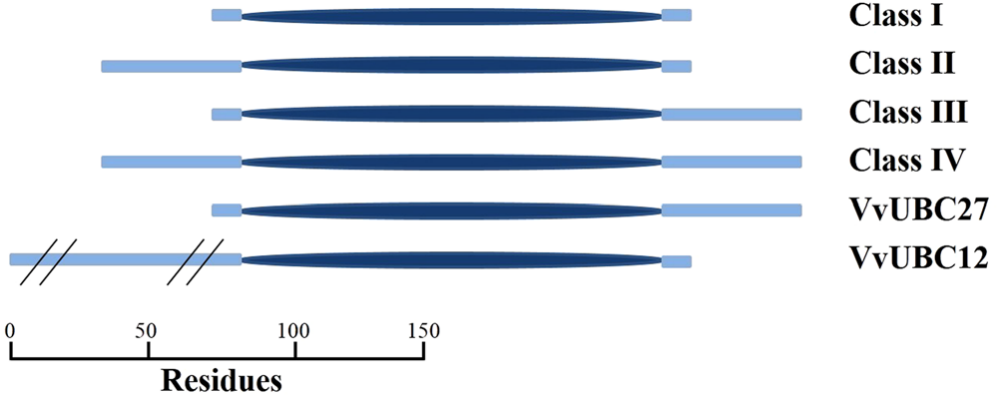
The domain architechture analysis of VvUBC proteins in *Vitis vinifera*. The UBC domain is indicated as a dark-blue ellipse and extensions as wathet blue blocks. The UBA domain of VvUBC27 is indicated as a green block and the X8 domain of VvUBC12 as a red block. Scale bar indicates protein length (aa).

UBC enzyme E2 gene family possesses a highly conserved UBC domain. Similar to E2 of human, VvUBC members can be divided into four classes according to existence of additional extensions to UBC domain^29^ (Fig. 2). In the present study, 50 amino acid residues (or less than 50 amino acid but performing other structural domain) beside UBC domain were regarded as additional extension. Most VvUBCs (28 members) possess a single UBC domain and are categorized as Class I. Class II (three members) features an N-terminal extension, Class III (six members) presents a C-terminal extension, and Class IV (six members) exhibits both extensions (see Supplemental Fig. S1). Interestingly, two VvUBCs contain other domains except for UBC, VvUBC27 contains a ubiquitin-associated domain^30^ (UBA) at C-terminal, and VvUBC12 contains X8 domain^31^ at N-terminal.

### Conserved motifs, gene structure, and promoter analysis of *VvUBCs*

**Figure 3:**
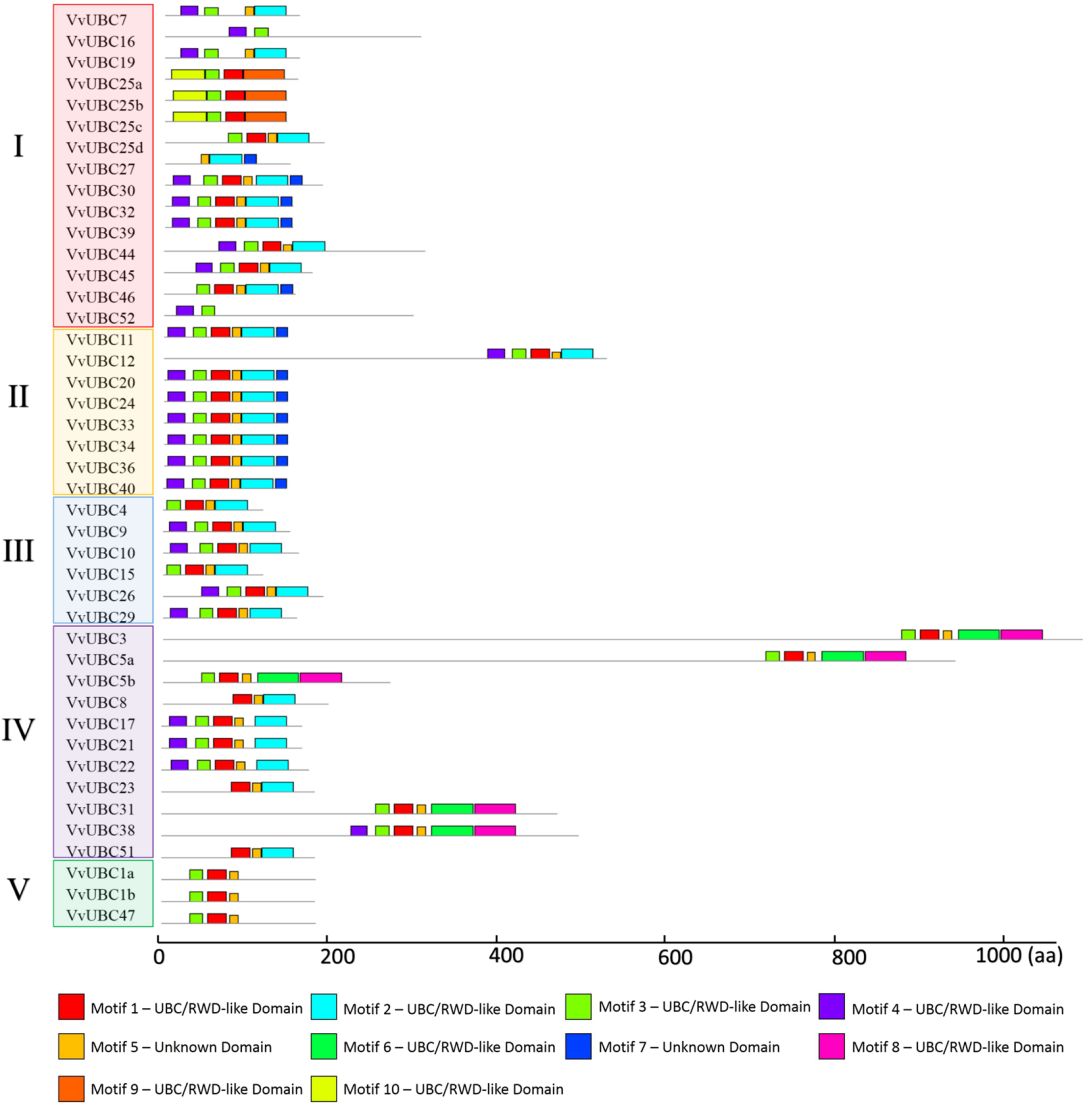
The conserved motifs analysis of 43 VvUBC members. The group was indicated by different color. Different motif was represented by box with different color. The legend of each motif were listed in Supplemental Figure S2.

Ten motifs were identified to illustrate VvUBC protein structure using MEME program and further annotated by InterPro Scan 5 (Fig. 3 and Fig. S2). Eight of 10 motifs (except motif 5 and motif 7) were localized within the UBC/RWD (RING finger-containing proteins, WD-repeat-containing proteins, and yeast DEAD or DEXD like helicases) domain, which contained an alpha-beta(4)-alpha(3) core fold, and was found in E2 and related proteins^32^, RING finger and WD repeat-containing proteins^33^, all VvUBCs contained at least two of them. VvUBC proteins contained 2–6 motifs, and length of motifs ranged from 11–50 amino acids (see Supplemental Fig. S1). Motifs 1 and 5 existed in almost all 43 VvUBCs except for five VvUBCs in Group I; by contrast, motifs 3, 2, and 4 existed in 39, 30, and 25 VvUBCs, respectively. The remaining motifs were detected in less than half of VvUBCs. Motifs 6 and 8 only existed in five VvUBCs in Group IV. Motifs 9 and 10 only existed in three VvUBCs in Group I. Group II and Group V respectively featured the same motifs except for VvUBC12.

Figure S3 shows gene structure of *VvUBC* genes. All *VvUBC* genes contained at least one untranslated region (UTR) in their 5′ or 3′ terminal and 3–11 exons. *VvUBCs* presented varying gene lengths ranging from 1267 bp (*VvUBC11*) to 23957 bp (*VvUBC44*). Length of coding sequence (CDS) averagely accounted for 10.45% of the whole gene length. This length did not relate to gene length.

Locations of promoter region compared to transcriptional initiation site range from −15000 bp (*VvUBC15*) to −115 bp (*VvUBC38*). Tween-three *VvUBCs* are located in the positive strand, whereas 20 *VvUBCs* are in the negative strand (Fig. S4).

### Chromosome localization and gene duplication analysis of *VvUBCs*

A total of 43 *VvUBCs* were distributed in all chromosomes except for chromosome 10, and most genes were close to chromosome terminal (Fig. S5). Chromosomes 6 and 8 contained the most *VvUBCs* (5 members), and other chromosomes contained 1–3 *VvUBCs*.

**Figure 4:**
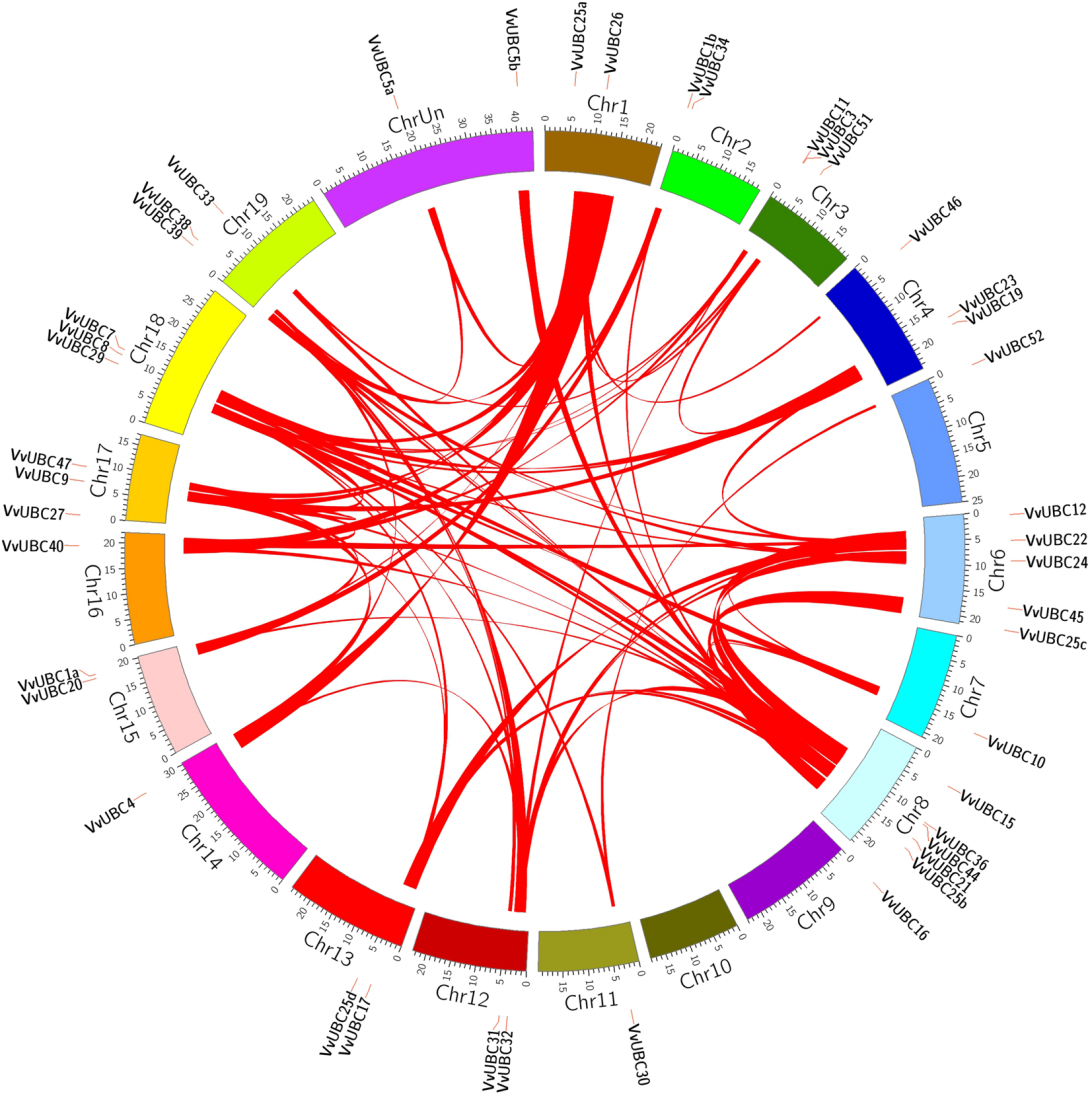
WGD related gene duplication analyses of *VvUBCs*. *VvUBCs* are indicated by vertical orange lines. Red bars denote syntenic regions. Chromosomes were indicated in different colors.

According to the whole genome duplication (WGD) related gene duplication analysis, WGD of *VvUBCs* occurred during grape genome evolution (Fig. 4), and a group of 36 *VvUBCs* were involved in 71 WGD events. For example, *VvUBC26* located in Chromosomes 1 and *VvUBC4* in Chromosome 14 are relative genes. These WGD events accounted for 83.72% (36 of 43) of *VvUBCs* gene expansion.

### Temporal and spatial expression patterns of *VvUBCs*

A total of 42 *VvUBCs* (without *VvUBC5b*) were identified by transcriptome analysis (GSE36128) of 54 organs in Corvina (*Vitis vinifera*) (Fig. S6). Expression of numerous *VvUBC*s showed significant changes during grapevine development. *VvUBC12* showed decreasing tendency in all organs (Fig. S6). During berry ripening, *VvUBC3* increased in three berry tissues (berry pericarp, berry flesh, and berry skin), whereas *VvUBC34* decreased, *VvUBC7* was up-regulated first and then down-regulated. In post-harvest withering stage, *VvUBC3* was rapidly up-regulated in three berry tissues (berry pericarp, berry flesh, and berry skin) and reached the highest level in post-harvest withering-III stage, whereas *VvUBC7/29/34* were down-regulated significantly. Several *VvUBCs* were expressed specially in different organs. For example, *VvUBC11* showed low expression level in berry but is highly expressed in leaves, especially in senescent leaves. *VvUBC45* featured higher expression level in winter buds than other organs.

**Figure 5:**
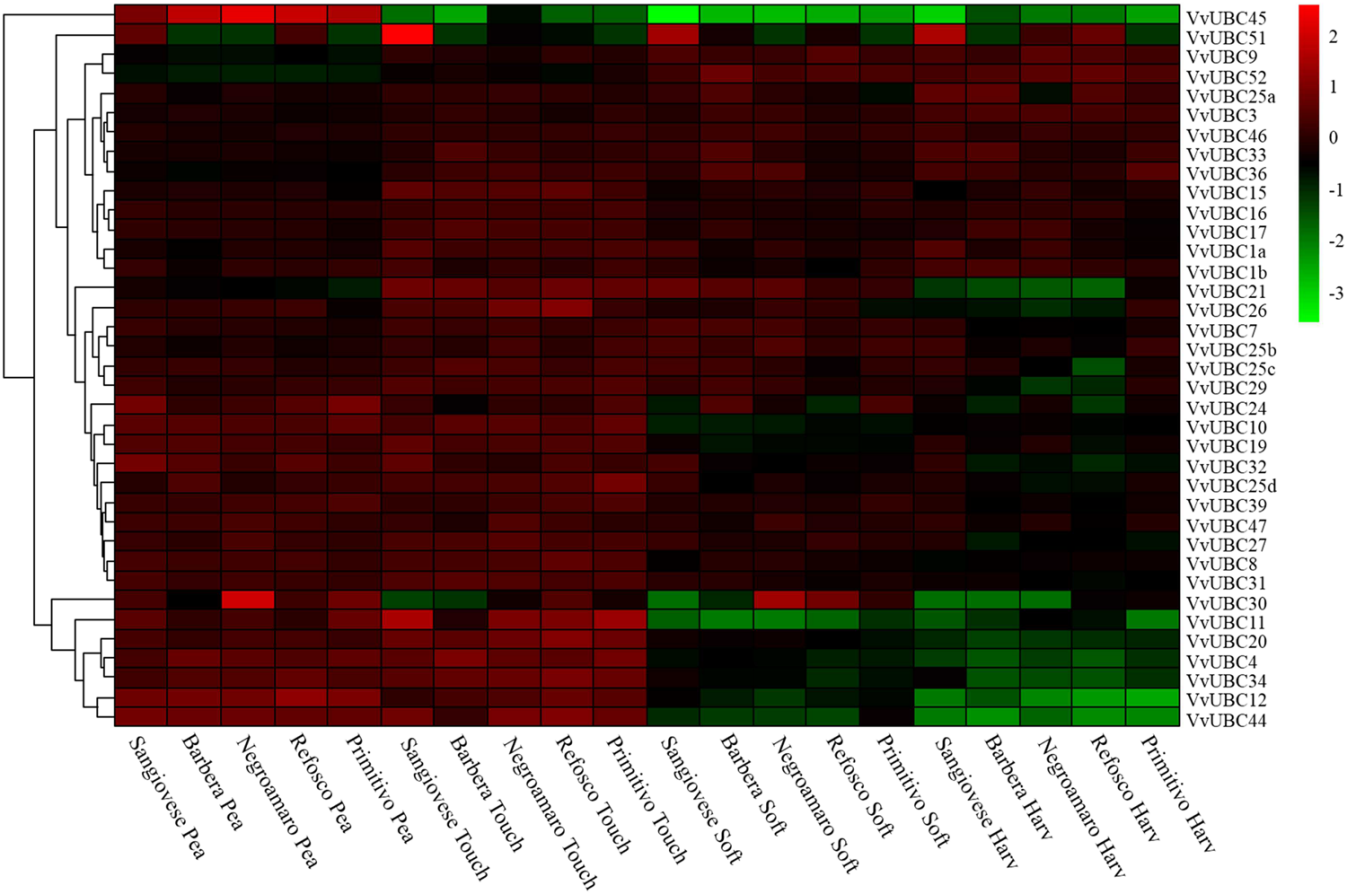
Expression analysis of *VvUBCs* in different periods among five species using GSE62744. The heatmap was performed by R. Blocks with different colors indicate the expression level relative to the expression average level, original data was normalized by calculate log2 value of the ratio of expression level to expression average level: higher than average(red), equal to average(black), lower than average(green).

To characterize expression pattern of *VvUBCs* in different genotypes, a transcriptome analysis in five varieties (Sangiovese, Barbera, Negroamaro, Refosco, and Primitivo) was performed using published data (GSE62744). This analysis was performed in four berry developmental stages (pea size, berry tough, soft, and harvest) (Fig. 5). Similar expression patterns of *VvUBCs* were observed in different varieties. Thirty-seven out of 43 *VvUBC* genes were expressed in berries. Most genes showed increasing or decreasing expression levels during ripening. Six *VvUBCs* (*VvUBC4/11/12/20/34/44*) were remarkably down-regulated in the last two stages of ripening (soft and harvest). *VvUBC*21 was up-regulated at berry tough stage then down-regulated at soft stage. *VvUBC45* was significantly down-regulated after pea size stage. Interestingly, *VvUBC51* showed higher expression levels in Sangiovese than other four varieties. The other *VvUBCs* slightly decreased or increased during grape ripening.

**Figure 6:**
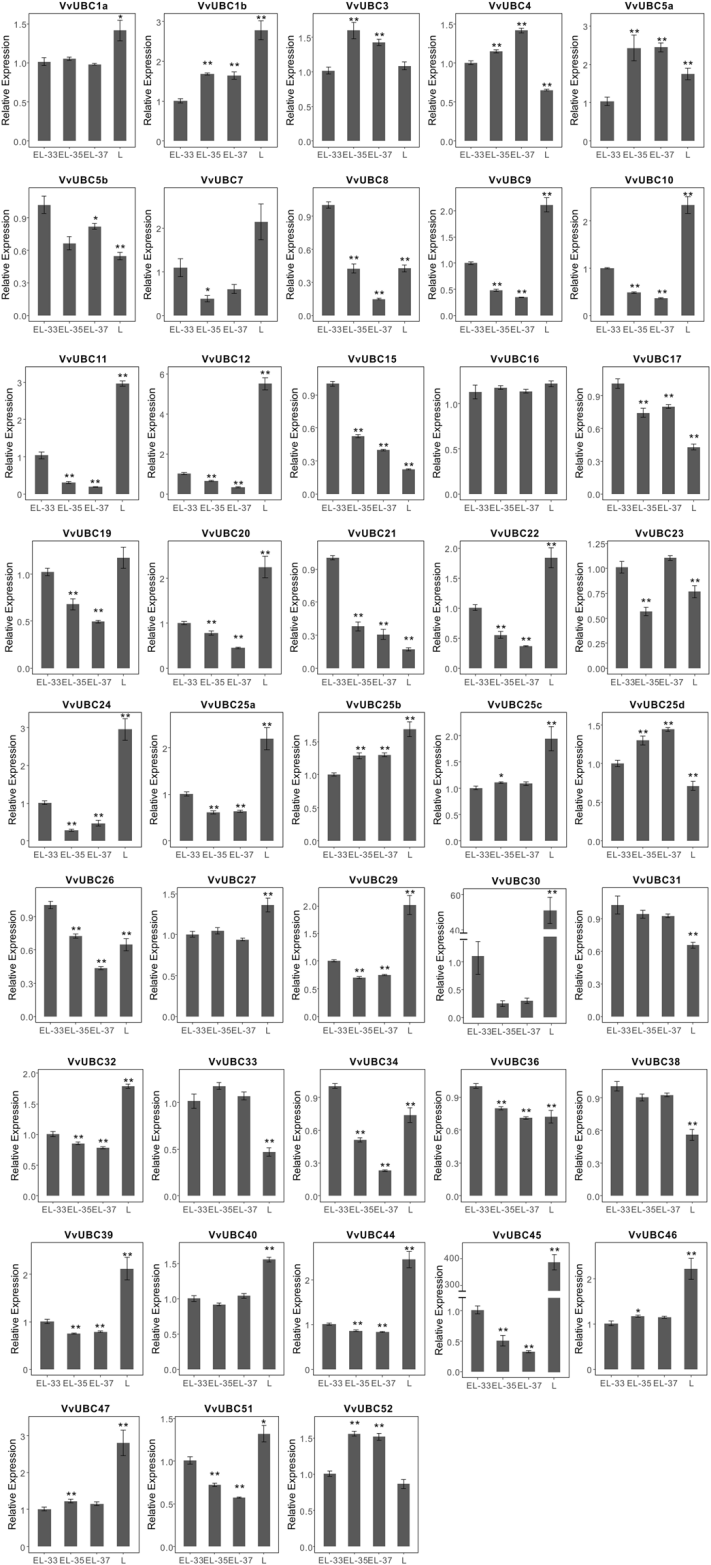
qRT-PCR results of 43 *VvUBCs* in young leaves and berries. EL-33, EL-35, EL-37 represent three ripening stage indicating by previous study^34^. L present young leaves. Data was normalized to *VvActin* gene expression level. Each *VvUBCs* at EL-33 stage was normalized as “1”. The mean expression value was calculated from three independent replicates. Vertical bars indicate the standard error of mean. **P < 0.01 and *P < 0.05 compared with berries in EL-33 stage.

To compare the expression patterns in leaves and during fruit ripening, all 43 *VvUBCs* were performed qRT-PCR using Cabernet Sauvignon. Most *VvUBCs* (22 of 43) showed down-regulated in three stage^34^ (EL-33, EL-35, EL-37), especially in *VvUBC8* and *VvUBC11*, which were down-regulated more than 5 fold at EL-37 stage compared to EL-33 stage (Fig. 6). Nine *VvUBCs* were up-regulated, and three *VvUBCs* (*VvUBC7/23/24*) deceased at veraison stage (EL-35) then increased at EL-37 stage. The transcript level of *VvUBC3* peaked at veraison stage then declined until EL-37 stage. Eighteen of 43 *VvUBCs* showed higher expression level in young leaves than that in berries, especially *VvUBC30* and *VvUBC45*, which approximately were 50 and 400 fold in leaves compared to berries, respectively. Eleven *VvUBCs* showed lower expression level in young leaves than that in berries (Fig. 6).

### Expression analysis of response of *VvUBCs* to different abiotic stresses

Expression pattern of grape *UBC* genes under cold and heat stress were investigate using published data SRP018199 and GSE41423 respectively (Fig. S7). Seven (*VaUBC3/12/15/17/25d/31/33*) and six members (*VvUBC9/10/11/20/27/52*) responded to cold and heat treatments, respectively. In leaves of *Vitis amurensis Rupr*., four *VaUBCs* (*VaUBC3/25d/31/33*) were significantly down-regulated, and the other three *VaUBCs* were significantly up-regulated after 4 hours under 4 °C cold treatment. Heat treatment was performed under 45 °C using Cabernet Sauvignon, and then recovery at control condition. Four *VvUBCs* (*VvUBC9/10/20/52*) were detected obviously up-regulated compared with control after heat treatment in leaves, whereas *VvUBC11/27* was down-regulated slightly. When recovery after heat treatment, *VvUBC9/20/52* showed higher expression in control than treatment groups, whereas *VvUBC10/11/27* showed lower. Thirty-three *VvUBCs* were identified in Cabernet Sauvignon from GSE31677, but these genes show no remarkable change during 16 days of salt or water-deficit stress (Fig. S8).

**Figure 7:**
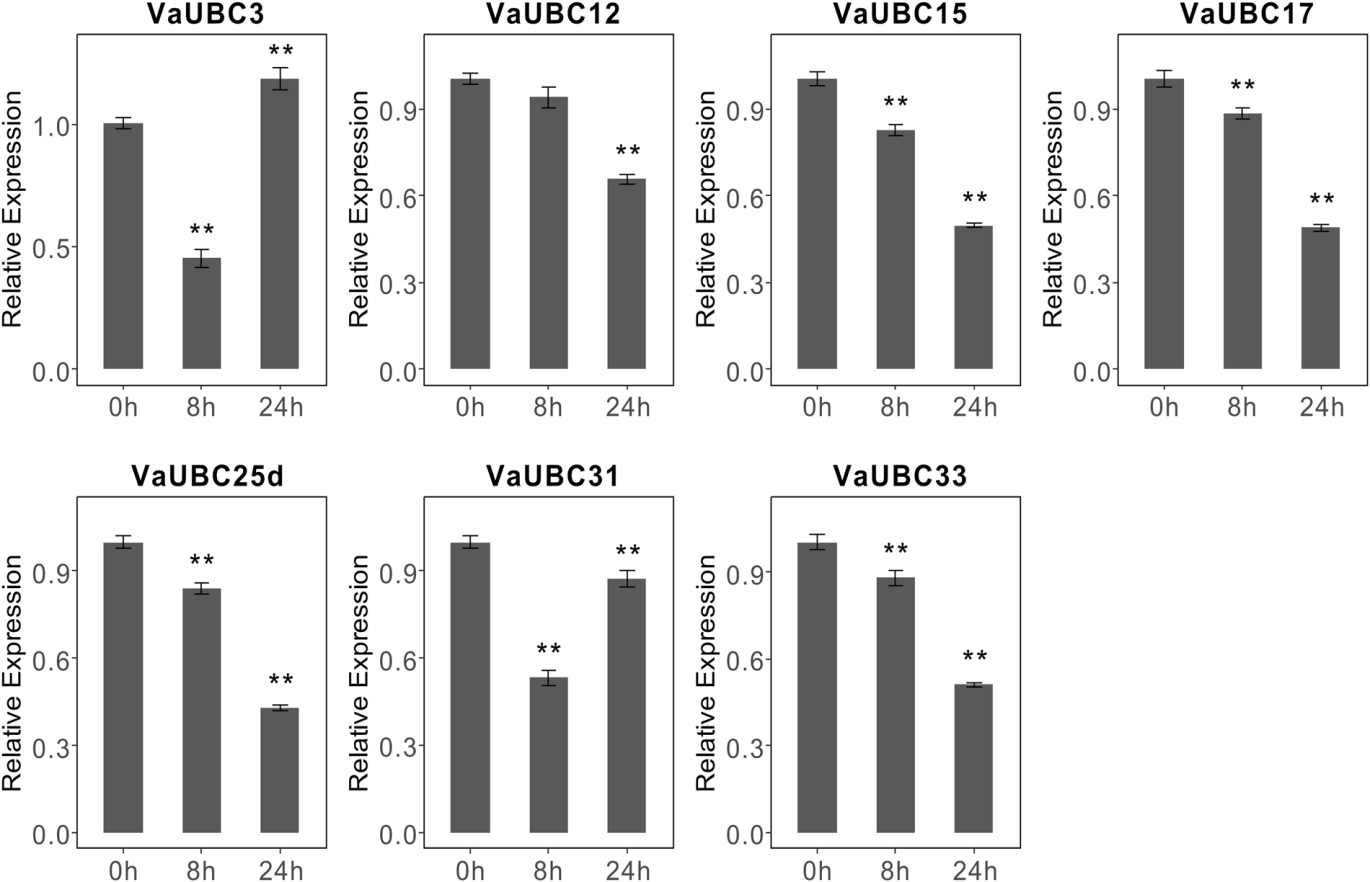
qRT-PCR results of seven *VaUBCs* under cold treatment. Data was normalized to *VvActin* gene expression level. Each *VaUBCs* at 0 h was normalized as “1”. The mean expression value was calculated from three independent replicates. Vertical bars indicate the standard error of mean. **P < 0.01 and *P < 0.05 compared with 0 h.

**Figure 8:**
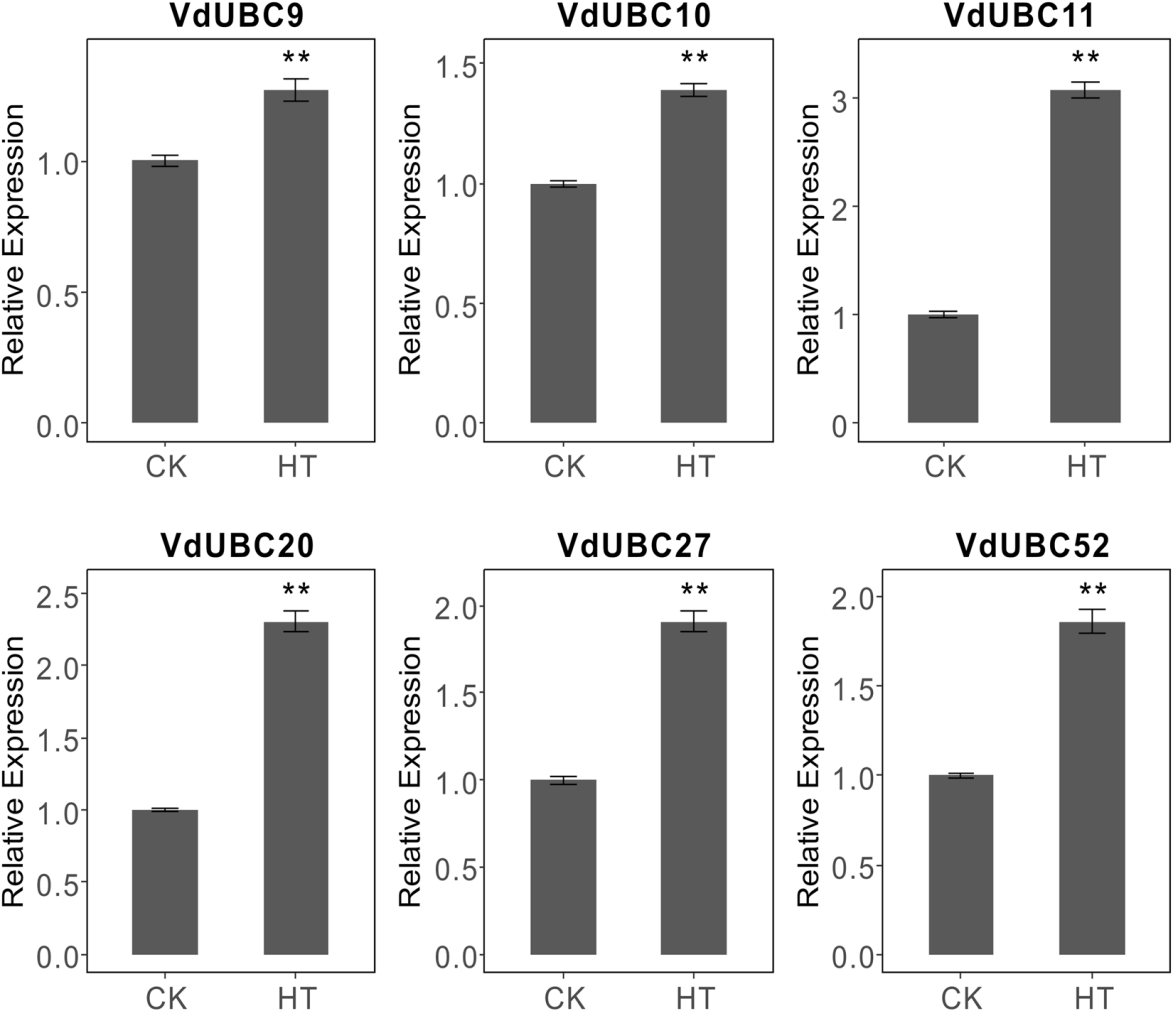
qRT-PCR results of six *VdUBCs* under heat treatment. Data was normalized to *VvActin* gene expression level. CK and HT represent the control and heat treatment, respectively. Each *VdUBCs* in CK was normalized as “1”. The mean expression value was calculated from three independent replicates. Vertical bars indicate the standard error of mean. **P < 0.01 and *P < 0.05 compared with CK.

To confirm the transcriptome results, these *UBCs* of grape were performed qRT-PCR (Fig. 7 and Fig. 8) in *Vitis amurensis* and *Vitis davidii*, respectively. Four *VaUBCs* (*VaUBC15/17/25d/33*) continuously decreased during cold treatment (4 °C,0–24 h), *VaUBC3* and *VaUBC31* decreased at 8 h and then increased at 24 h, *VaUBC12* showed no significant change at 8 h, but decreased at 24 h (Fig. 7). Six *VdUBCs* (*VdUBC9/10/11/20/27/52*) were up-regulated in detached leaves after heat treatment (38 °C 2 h, 47 °C 40 min, Fig. 8).

## Discussion

In this study, 43 *VvUBCs* were identified in grape, and this number was higher than the 39 discovered in rice^15^, 41 in Arabidopsis^14^, less than 52 in tomato^21^, 50 in human^13^, and 75 in maize^16^. These *VvUBCs* were divided into five groups based on a phylogenetic tree. VvUBC proteins contained almost similar motifs in one group, especially in Groups II and V. CDS length accounted for 10.45% of the whole gene length, and their promoter location showed a large difference. Chromosome location showed an uneven distribution of *VvUBC* genes in 19 chromosomes, but no *VvUBC* gene was found in chromosome 10. Whole genome duplication events played a significant role in evolution of many organisms^35^, complex WGD events existed in *VvUBCs*, indicating that *VvUBCs* perform various functions during grape development. All these analyses showed large differences between *VvUBCs* in protein structure, gene structure, and promoter location. *VvUBCs* in one group may exhibit relative functions.

According to domain analysis, conserved domain UBC exists in all VvUBCs. Protein structure of E2 genes is uncommon in plants, but it is widely used in human research. In humans, E2s can be classified based on existence of additional extensions aside from the UBC domain^29^. These extensions result in functional diversity of E2 genes; this functional diversity is related to subcellular localization and interaction between E2 and E3^36–40^. In this study, fifteen VvUBCs contained extensions with different roles. N-terminal of VvUBC12 contained an X8 domain and a transmembrane domain. This situation indicates that VvUBC12 may contribute to binding of carbohydrates^31^. Similar to UBE2K in humans^29^, VvUBC27 contained a UBA domain in C-terminal; this UBA domain might be related to ubiquitin binding^30^. However, direct role of UBA remains unclear. Conserved motif analysis showed that all VvUBCs contained at least two UBC/RWD domain motifs whereas consist of different motifs, indicating the VvUBCs identified in this study had conserved features of the E2 genes family, and they might play different function in ubiquitination process.

To gain deeper understanding of putative function of *VvUBC*, temporal and spatial expression profiles were analyzed. In tomato, E2 genes play an important role in regulation of fruit ripening, as determined by virus-induced gene silencing assay^21^. In grape, most *VvUBCs* in five Italian varieties change during ripening (Fig. 5), similar expression patterns of *VvUBCs* were obtained from qRT-PCR in Cabernet Sauvignon (Fig. 6), which indicating that E2 gene family might play extensive roles in grape ripening. *VvUBC45* showed different expression profiles in Sangiovese (Fig. 5), indicating its distinct roles in this fruit. Additionally, in Corvina, *VvUBC3/7/12/34* were rapidly up-regulated or down-regulated during grape berry development (Fig. S6), indicating involvement of these genes in fruit ripening. Interestingly, *VvUBC3* was up-regulated significantly in post-harvest withering stage and *VvUBC7/29/34* down-regulated (Fig. S6), they may play significant roles in post-harvest physiology. Aside from berries, E2 genes also played various roles in other organs. In *Arabidopsis*, *AtUBC22* participates in female gametophyte development^41^. *AtUBC1* and *AtUBC2* are ubiquitously expressed in roots, leaves, flowers, and seedlings and activation of FLOWERING LOCUS C allow these genes to repress flowering^42^. In Corvina, *VvUBC11* and *VvUBC45* exhibited high expression levels in senescing leaves and winter buds, respectively (Fig. S6), *VvUBC30* and *VvUBC45* showed high expression in young leaves of Cabernet Sauvignon (Fig. 6). These genes may play different roles in grape development compared with other *VvUBCs*.

E2 genes from both *Arabidopsis* and rice were not reported to be induced under cold stress^43^. The present study revealed that *ZmUBCs* changed significantly under cold conditions^16^. In *Vitis amurensis*, seven *VaUBCs* (*VaUBC3/12/15/17/25d/31/33*) responded to cold treatment (Fig. S7), and the results were confirmed by qRT-PCR (Fig. 7), but the change tendency of *VaUBCs* showed a slight difference, which might because of the different cold treatment time. At present, no E2 genes were reported to involve in heat stress. However, expression levels of six *UBC* genes were obviously changed under heat condition and recovery condition from heat treatment in not only RNA-seq data (Fig. S7) but also qRT-PCR (Fig. 8) in grape in this study. The results indicated that these grape *UBC* genes might be involved in heat response mechanism in grapes. E2 genes presented different responses to heat and cold stresses. These results indicated that there might be different regulatory mechanisms of ubiquitination in response to heat and cold stresses.

E2 genes in several species were functional under salt or drought. *GmUBC2* showed enhanced drought and salt tolerance in soybean^18^, whereas *AtUBC32* was strongly induced by salt stress in *Arabidopsis* ^20^. Three genes (*OsUBC13/15/45*) were also up-regulated under salt and drought stresses in rice^15^. In peanut plants, the physiological water stress induced by polyethylene glycol, high salinity, abscisic acid, or low temperature, changed the expression levels of *AhUBC2* ^43^. Increased transcript levels of *CmUBC* were observed during drought and salinity stresses in *Cucumis melo* ^44^. Based on previous transcriptome resources, *VvUBCs* showed no significant changes in response to drought and salt stresses in grapes (Fig. S8). This result indicated that E2 genes may play different roles in herbaceous and woody plants.

## Conclusion

In this study, 43 *VvUBC* members were identified and divided into five groups based on their phylogenetic tree. Protein and gene sequences, and duplication events were analyzed to predict functional characteristics of *VvUBC* genes. Transcriptome data and qRT-PCR results presented significant roles of *VvUBCs* in grape growth, maturity and post-harvest physiology. Additionally, seven and six *VvUBCs* showed responses to cold and heat stresses, respectively. These responses may contributed to grape resistance mechanism. These results provide new insights into the E2 genes family in woody plants and a solid foundation for further research on grape breeding.

## Materials and Methods

### Identification of grape E2 family members

Tomato E2 family members were obtained from a previous research^21^, which was used in BLAST search to obtain candidate genes of E2 family in grapes. All protein sequences were obtained from the National Center for Biotechnology Information (NCBI) (http://www.ncbi.nlm.nih.gov). HMM was constructed using sequence data and was used to search UBC proteins in grapes with a cut-off E-value of 0.001. Then, results of BLAST and HMM searches were merged. Next, candidate UBC protein sequence was scanned again using the domain analysis tool NCBI-Conserved Domain Database (http://www.ncbi.nlm.nih.gov/Structure/cdd/wrpsb.cgi). Finally, 43 UBC proteins were identified in grapes.

### Phylogenetic analysis

A phylogenetic tree was generated by MEGA 6.0. Protein sequence was aligned by Clustal W. Then, alignment was imported into the MEGA 6.0 software, and phylogenetic tree was constructed using neighbor-joining statistical method with 1000 bootstrap replication.

### Analysis of conserved domain, conserved motif, gene structure and promoters

According to obtained VvUBC protein sequence, domain analysis of proteins was performed by SMART (http://smart.embl-heidelberg.de/). Then, conserved motifs were analyzed by MEME program (http://meme-suite.org/tools/meme). Furthermore, the motifs obtained were annotated using InterProScan (http://www.ebi.ac.uk/Tools/pfa/iprscan/). Promoter 3.0 was used to annotate grape genome to select the most suitable promoter of *VvUBCs*, illustrations of promoter and genes were constructed by Gene Structure Display Server (GSDS) software^45^ (http://gsds.cbi.pku.edu.cn/). Introns and exons of *VvUBCs* were detected in grape genomic annotation, and the diagram was constructed by GSDS^45^. Localization of *VvUBCs* in chromosome was determined according to grape genomic annotation, and diagram was generated by Mapchart 2.3.

### Gene duplication

Protein sequence in grape was used for self-BLAST search. Then, BLAST results and documented annotation were combined to analyze duplication of *VvUBCs* by MCscanX. Finally, a map was drawn by Circos.

### Plant growth and Treatments

To analyze expression of *VvUBC* genes in different tissue and fruit ripening, young leaves and berries were sampled from Cabernet Sauvignon (*Vitis vinifera*), which planted at the Germplasm Repository for Grapevines in the Institute of Botany of the Chinese Academy of Sciences, Beijing, China (39° 54′N, 116° 23′E). The vines were planted in 2007 in south-to-north oriented rows, trained to a fan-shape trellis with single trunk, and subjected to similar management practices for irrigation, fertilization, soil management, pruning, and disease control. Berries were sampled at three developmental stages (EL-33, EL-35, EL-37) according to EL system^34^, each sample was collected from nine clusters, and approximately 20 berries from three clusters formed one biological replicate. Sixth leaves were sampled with three biological replicates.

*Vitis amurensis* were used for cold treatment. Tissue cultured *Vitis amurensis* were grown on half-strength Murashige and Skoog (1/2 MS, pH 5.8) solid medium 1% sucrose and 0.7% agar in conical flasks (120 mL) in a growth chamber at 26 °C under a 16-h light/8-h dark photoperiod and 100μmol m^−2^ s^−1^ light intensity. Six-week-old plantlets were subjected to cold stress, plantlets were transferred to a low-temperature chamber at 4 °C with a 16-h light/8-h darkness cycle. The shoot apex with the first fully expanded leaf was harvested at specific time (0 h, 8 h, and 24 h) after initiating the treatments with three biological replicates.

Spine grape (*Vitis davidii*) was used to analyze expression of *VvUBC* genes under heat stress. The vines were planted in the same condition as Cabernet Sauvignon introduced above. Detached leaves of approximately 30 days in age were used for heat treatment according to previous study^46^. In June of 2017, samples were taken in the morning, placed in the dark with the petiole in water, and then treated by heat stress. The heat stress process was as follows: leaf discs (5.5 cm in diameter) were cut from the detached sample leaves, wrapped in a wet paper towel and placed in a small vessel made of aluminum foil. The vessels were then floated on water in a temperature-controlled water bath, 38 °C 2 h and then 47 °C 40 minutes. Leaves samples were collected with three biological replicates at this time. The control was the same condition as heat treatment except temperature-controlled water bath in 25 °C, and the leaves samples were collected at the same time with three biological replicates.

### RNA extraction and quantitative real-time PCR (qRT-PCR) analysis

All samples were immediately obtained frozen in liquid nitrogen and stored at −80 °C for RNA extraction. Total RNA was extracted from collected samples using RNAprep Pure Plant Kit (TIANGEN, Beijing, China) following the manufacturer’s procedure. A maximum of 1 μg total RNA was used for synthesizing cDNA by HiScript Q RT SuperMix (Vazyme, Nanjing, China), and the product was subjected to qRT-PCR with an Opticon thermocycler (CFX Connect Real-Time System; Bio-Rad, Hercules, CA) using SYBR Green PCR master mix (Vazyme, Nanjing, China) according to the manufacturer’s instructions. The PCR cycling conditions were as follows: 95 °C for 10 min, 40 cycles of 95 °C for 10 s, 60 °C for 30 s; a 65–95 °C melt curve was analyzed to detect possible primer dimers or nonspecific amplification. *VvActin* (Accession number: EC969944) was used as stable reference genes. Gene specific primer pairs for qRT-PCR (listed in Table S1) were designed by NCBI Primer BLAST. The specificity of the primers was further verified through gel electrophoresis and reaction product sequencing. Three biological replicates were performed to ensure the accuracy of results. The relative expression of the target genes was determined using the 2^−ΔΔCt^ method^47^. All experiments were performed with three biological replicates and three technical replicates. Statistical difference were performed by t-test (**P < 0.01, *P < 0.05, n = 3) using R software.

### Transcriptomic resources

Transcriptomic data used in this study were obtained from previous research^48–52^. Expression levels in different organs were analyzed using GSE36128^48^. A total of 54 organs were collected from grapevines Corvina (*Vitis vinifera*) for RNA extraction. The entire list of 54 organs can be found as Supplementary Table S2. Three biological replicates were obtained for each sample. Data of four stage of berries in five varieties analysis were obtained from GSE62744^49^. Grape berries were collected from five red-skin grapevine (*Vitis vinifera*) cultivars (Sangiovese, Barbera, Negro amaro, Refosco, and Primitivo) at four phenological stages (pea size, berry tough, soft, and harvest), with three biological replicates acquired for each sample.

Cold treatment in SRP018199 was performed as follows^50^: *Vitis amurensis* seedlings were grown in 16 h light/8 h dark photoperiod at 26 °C. These seedlings were then transferred into a chamber at 24 °C under 16 h light at 6:00 am. Cold treatment was started at 9:00 am with constant light. During the first four hours, temperature dropped to 5 °C per hour and was held at 4 °C for an additional four hours. Seedlings used for control were also transferred to growth chambers but without cold treatment. Shoot apices with one well-developed leaf were harvested from three independent replicates. RNAs were isolated for digital expression library construction.

Heat treatment in GSE41423 was conducted as follows^51^: Cabernet Sauvignon (*Vitis vinifera*) was grown in 25/18 °C day/night condition before treatment. Then, the experimental group was treated at 45 °C from 9:00 to 14:30. Next, leaf samples were obtained and recovered rapidly at 25 °C for 15 min. Leaf samples were collected the following morning at 9:00. Control group was grown in 25/18 °C day/night condition. Leaf samples were collected from the experimental group.

Cabernet Sauvignon (*Vitis vinifera*) were treated under water-deficit and salinity stress conditions (GSE31677)^52^. This process is listed in Supplemental Table S3.

### Data Availability

The datasets analysed during the current study are available from the corresponding author on reasonable request.

## Electronic supplementary material

Supplementary Information
