# Supplementary material for: Involvement of Ubiquitin-Conjugating Enzyme (E2 Gene Family) in Ripening Process and Response to Cold and Heat Stress of Vitis vinifera
Source: Sci Rep. 2017 Oct 16;7:13290. doi: 10.1038/s41598-017-13513-x (PMC5643510; doi:10.1038/s41598-017-13513-x)
Supplement: Supplementary file 1 — Supplementary Information [file 41598_2017_13513_MOESM1_ESM.doc]

**Involvement of Ubiquitin-Conjugating Enzyme (E2 Gene Family) in Ripening Process and Response to Cold and Heat Stress of *Vitis vinifera***

**Yingying Gao^1, 2, 3^, Yi Wang^2, 3^, Haiping Xin^1^, Shaohua Li^2^, Zhenchang Liang^2, 4^**

**
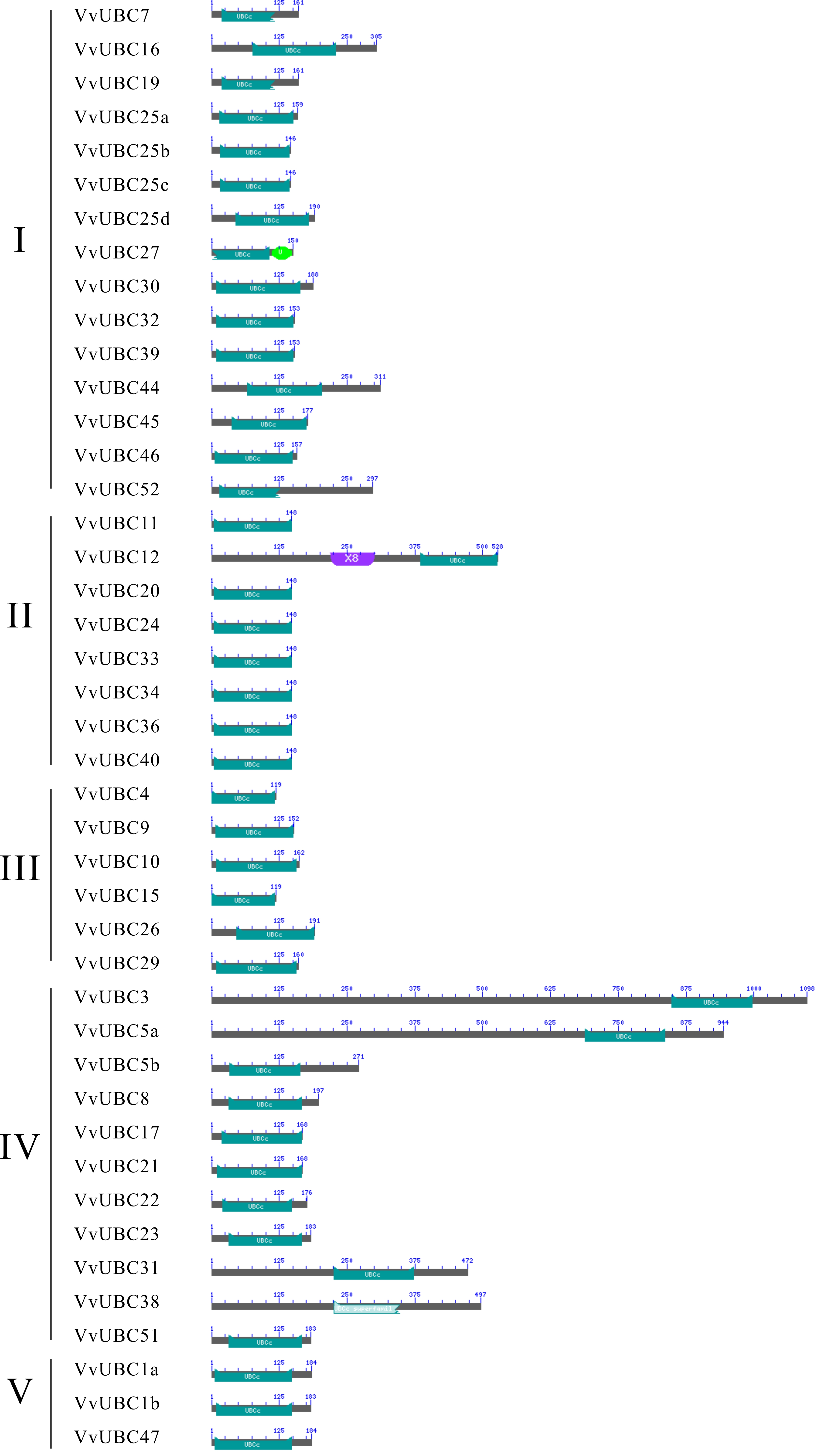
**

**Supplemental Figure S1. The conserved domain analysis of 43 VvUBCs.** UBC domain was shown in blue strip. UBA domain of VvUBC27 and X8 domain of VvUBC12 were shown by different colors.

**
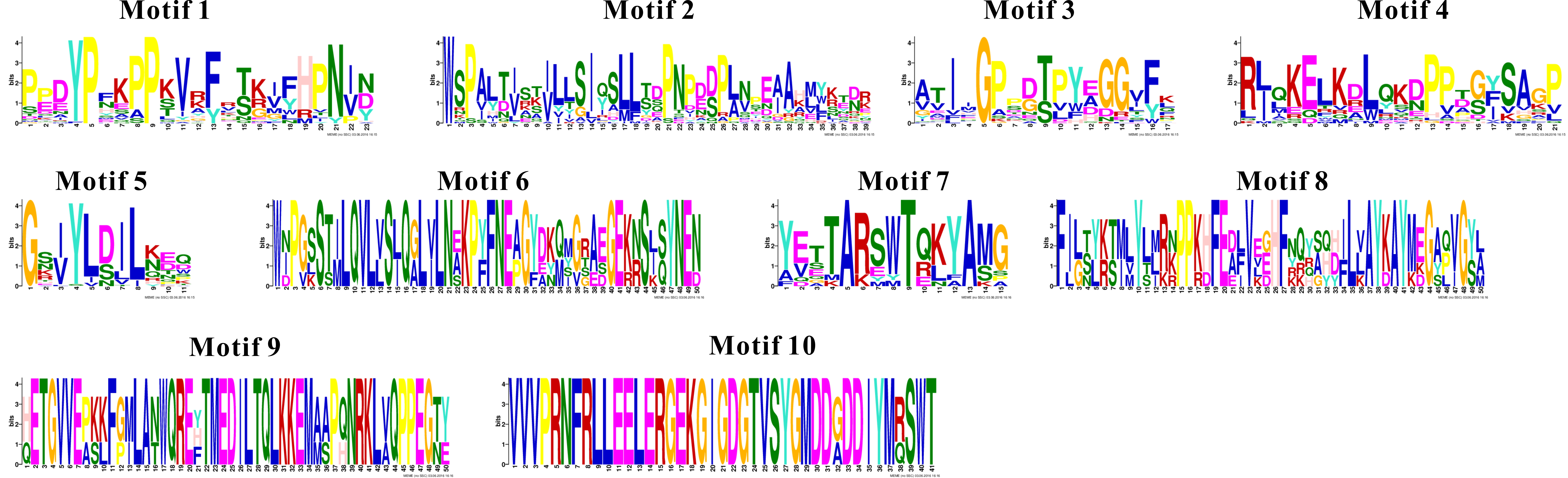
**

**Supplemental Figure S2. The conserved motif sequence of VvUBCs.** The legends were generated in MEME program.


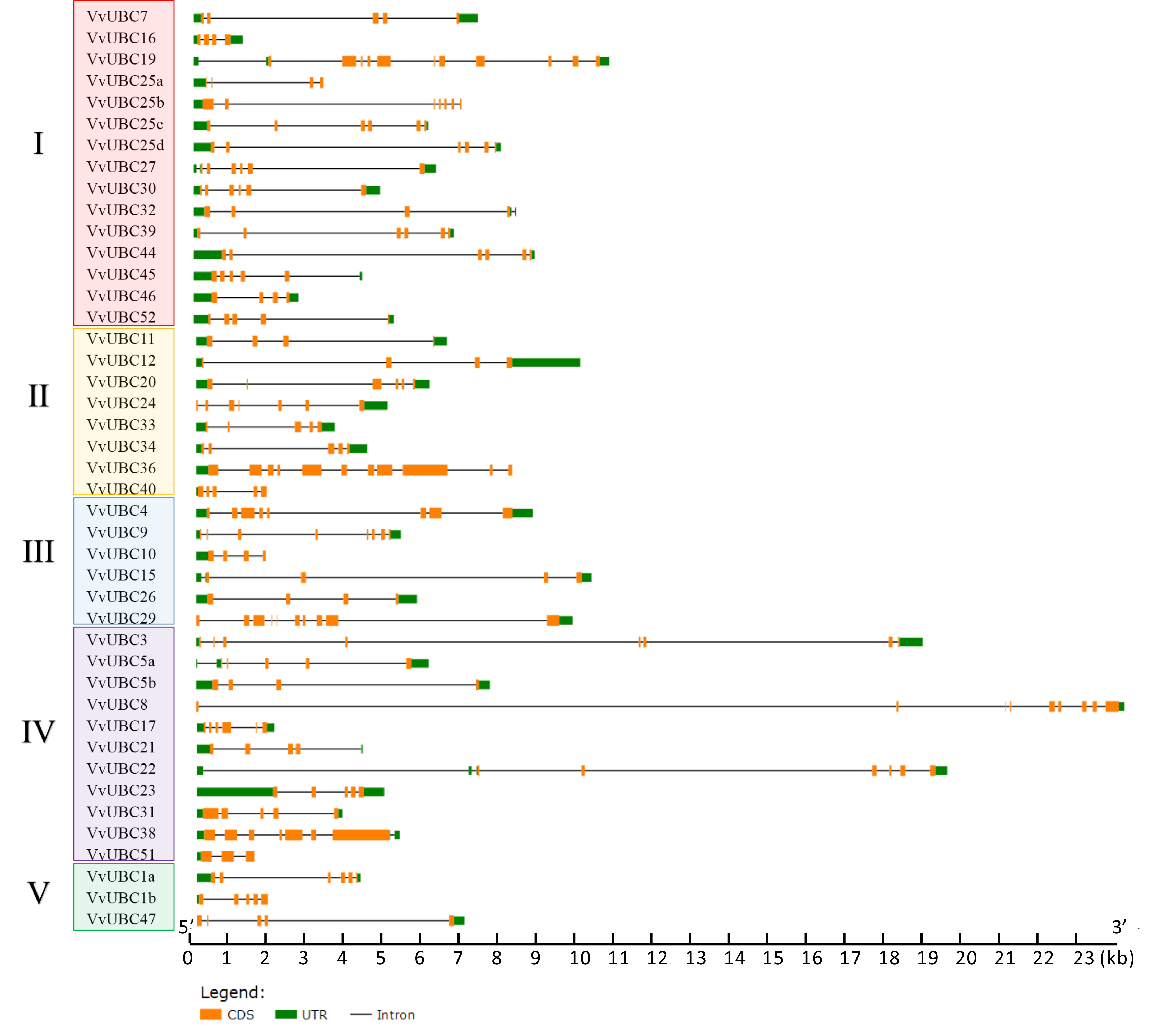


**Supplemental Figure S3. The gene structure analysis of 43 *VvUBCs*.** The group was indicated by different color. The blue thick line and yellow thick line represent UTR and CDS, respectively. The black line between thick line represent intron.


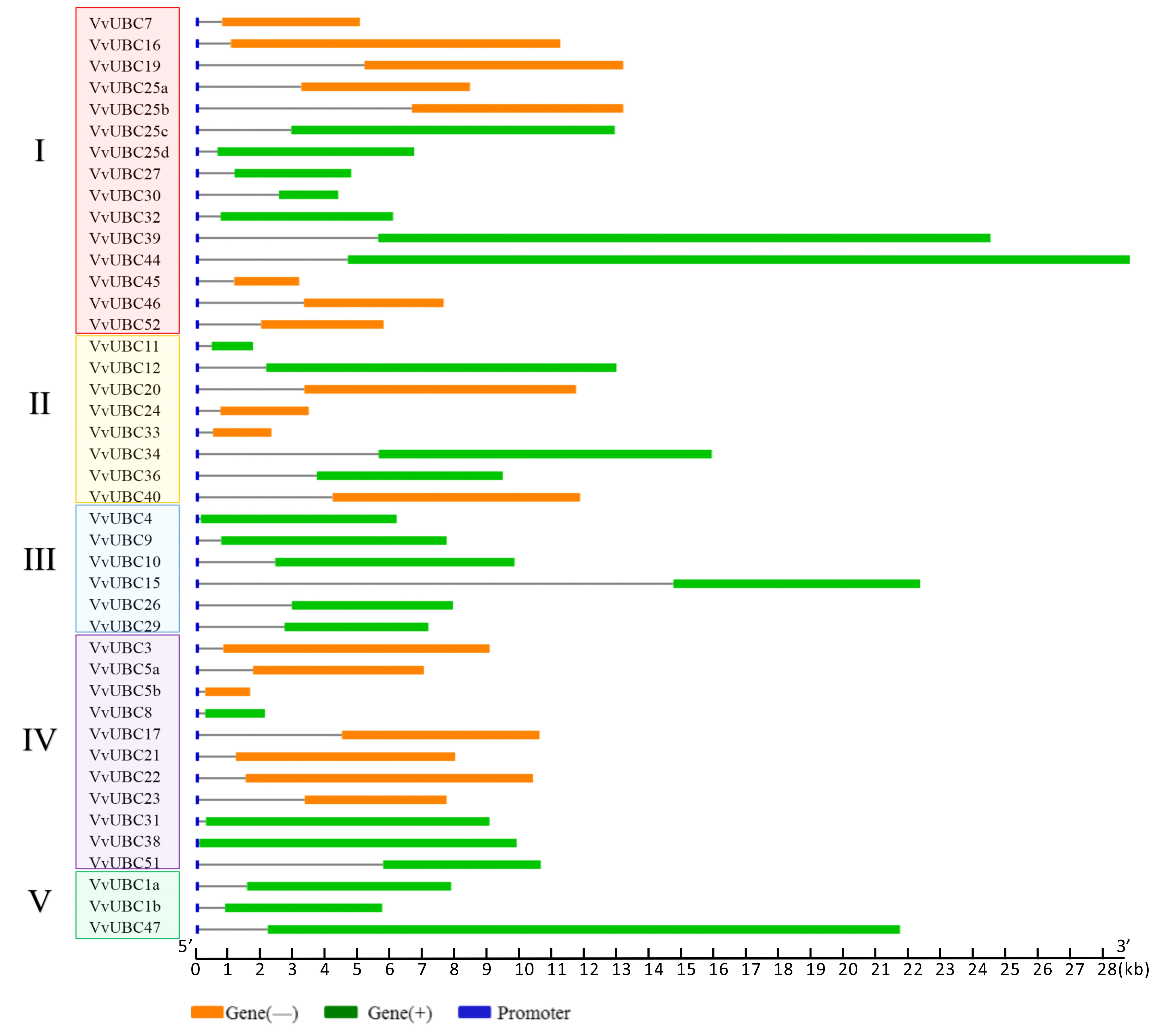


**Supplemental Figure S4. The promoter analysis of 43 *VvUBCs*.** The group was indicated by different color. The blue thick line represents the start location of promoter, the *VvUBCs* in plus strand and minus strand were represented by orange and green thick line, respectively.





**Supplemental Figure S5. Location and distribution of *VvUBCs* on the grape chromosomes.** The vertical columns represent chromosomes with the gene names shown on the right.

**
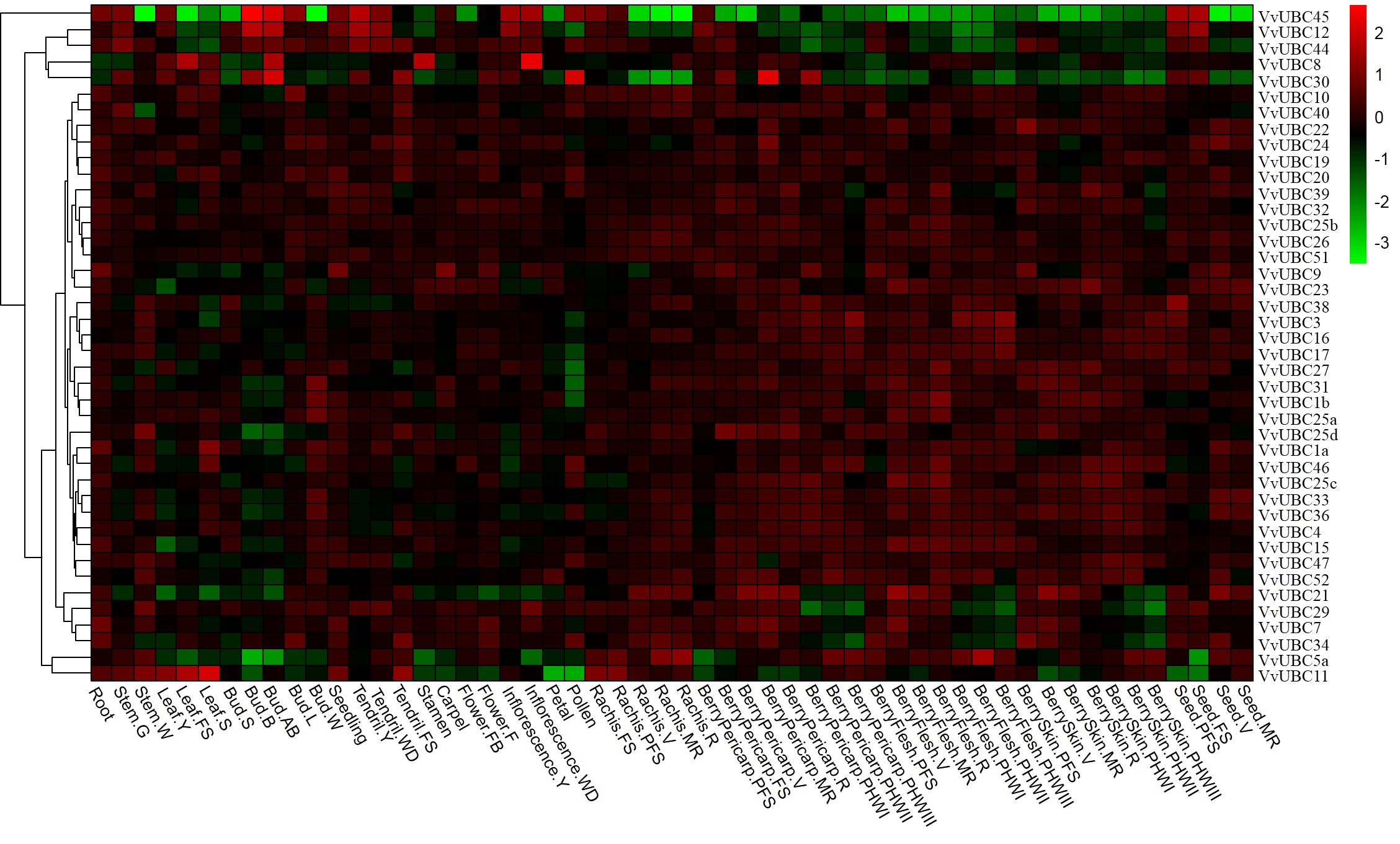
**

**Supplemental Figure S6. Expression analysis of 42 *VvUBCs* in different organs using GSE36128.** The heatmap was performed by R. Original data were normalized by calculating log2 value of the ratio of expression level to expression average level. Blocks with different colors indicate the expression level relative to the expression average level: higher than average (red), equal to average (black), lower than average (green). Different organs were indicated by abbreviation^48^ (Supplemental Table S2). **
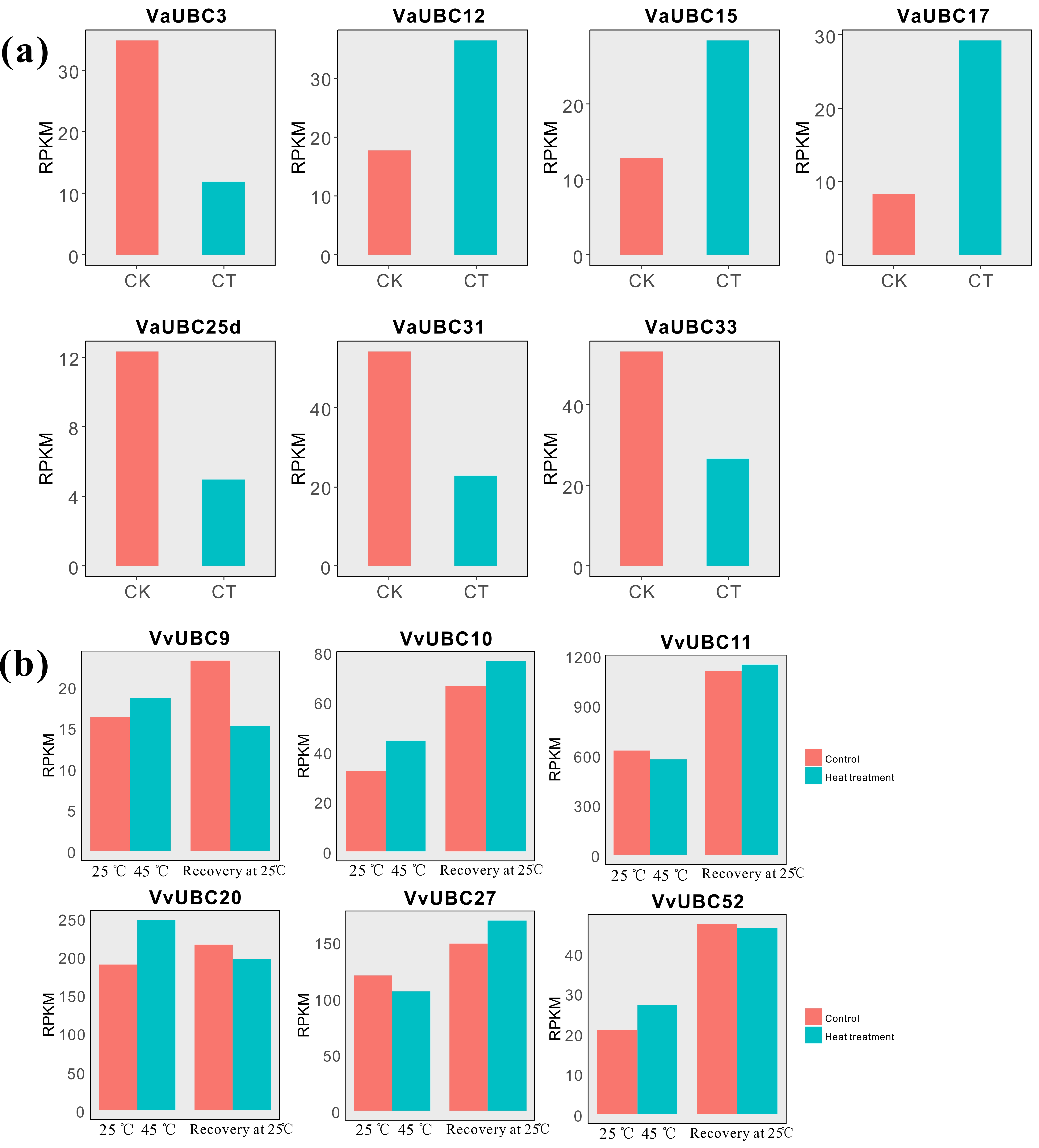
**

**Supplemental Figure S7. Expression analysis of grape *UBC* genes under cold (a) and heat (b) stress.** Transcriptome data were used to perform bar graph with R software. (a) The data was obtained from SRP018199. CK and CT represent control and cold treatment, respectively. (b) The data was obtained from GSE41423.Control and heat treatment were indicated by bars in different colors, x-axis showed the temperature in heat treatment and recovery.

**
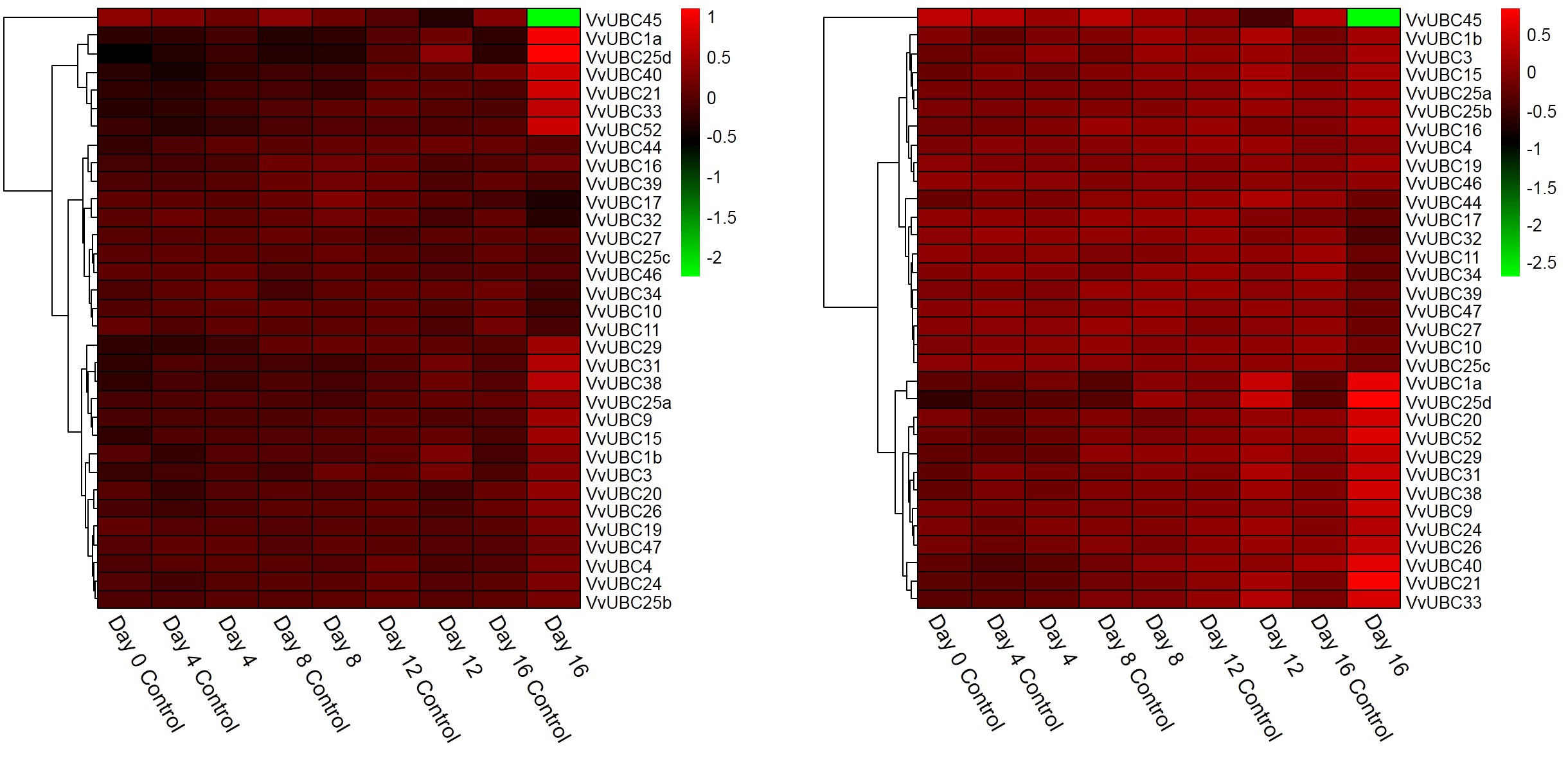
**

**(a)**

**(b)**

**Supplemental Figure S8. Expression analysis of *VvUBCs* under water-deficit (a) and salt stress (b) using GSE31677.** The heatmap was performed by R. Original data were normalized by calculating log2 value of the ratio of expression level to expression average level. Blocks with different colors indicate the expression level relative to the expression average level: higher than average (red), equal to average (black), lower than average (green).

**Supplemental Table S1. The primer sequence for qRT-PCR**

| *VvUBC1a-F* | AGGTGTTCCTTCCGCAACTT | *VvUBC25b-F* | GCCACCAAGTGTCCGTTTTC |
| --- | --- | --- | --- |
| *VvUBC1a-R* | ATAAGCAGTTCGGTCACGCA | *VvUBC25b-R* | ACTTCTTAGGTTCCACCACTCC |
| *VvUBC1b-F* | CTTCTCTACCCCAACCCATCAG | *VvUBC25c-F* | CGTGGGGAGAAGGGTATTGG |
| *VvUBC1b-R* | GCAGCCCCAATGTTTTCTGG | *VvUBC25c-R* | CCTGTCCAAGACCGCATGTAT |
| *VvUBC3-F* | GGGAGGAAGTTGTAAGCGTCT | *VvUBC25d-F* | TTGGGGTATCTGCTTTCCCTG |
| *VvUBC3-R* | TTGTTTAACTGGGGACAACCGA | *VvUBC25d-R* | GGGTAGTCAAGGGGGAAACG |
| *VvUBC4-F* | TGCCGATGGAAGCATCTGTT | *VvUBC26-F* | GAATAAGCCACCAACTGTACGC |
| *VvUBC4-R* | AACATCCGTGCAGCTTCTGA | *VvUBC26-R* | GTCACAAAGCAGCGACTGGA |
| *VvUBC5a-F* | CTGCTCTGGTTGGTTCTGGT | *VvUBC27-F* | ATCAGCAGTCAAAGTGGAGC |
| *VvUBC5a-R* | ATACACCAATCTCCAAGCTGCC | *VvUBC27-R* | CATCAGGTTGAGGGGCAGAG |
| *VvUBC5b-F* | CTCACTACCCTGGTTCGCAG | *VvUBC29-F* | GACTACCCTAGCAAACCCCC |
| *VvUBC5b-R* | CGCAGGAGATCCATCCTTTCC | *VvUBC29-R* | CAGGTCTCCAACCACTGTCT |
| *VvUBC7-F* | TTCTTCGGGAGGACTGGAAC | *VvUBC30-F* | TTGACGCCCAGATTGAAGGA |
| *VvUBC7-R* | GAAGAAGCCCTCAACAGTGC | *VvUBC30-R* | TCGCAAAAGTCACACTCGGA |
| *VvUBC8-F* | ACCACCAACAGTGCGATTTG | *VvUBC31-F* | TACGCATCACAGTCTTCGCA |
| *VvUBC8-R* | CAGGAGAATTGGGGTTGGGA | *VvUBC31-R* | ATTTCCCCTTGTTCCCACCC |
| *VvUBC9-F* | CAGAGACGCTTCCAGATGGG | *VvUBC32-F* | GCTCCCAAGGTTCGCTTCTT |
| *VvUBC9-R* | GGAACTTGCATTTTGGGGGC | *VvUBC32-R* | TCAGCAACACCGTTCGGATT |
| *VvUBC10-F* | GCAAGCCCCCAAAATGCAAG | *VvUBC33-F* | GGACCCTCCCACTTCTTGC |
| *VvUBC10-R* | TAGCTGGTCTCCATCCGCTAT | *VvUBC33-R* | CAGAAACACACCTCCAGCGA |
| *VvUBC11-F* | TCCCGTGGCTGAGGATATGT | *VvUBC34-F* | CCACCAAAGGTTGCTTTCAGG |
| *VvUBC11-R* | CAGGTGGGAAATGGATGGTCA | *VvUBC34-R* | GGCTCCACTGCTCCTTCAAAA |
| *VvUBC12-F* | TGGAGGAAGATGTTGGCCTT | *VvUBC36-F* | GCTGCTCTCCATCTGCTCAT |
| *VvUBC12-R* | AGGGTGGAGACCCAGTGATAG | *VvUBC36-R* | TTCATACTTGGCTCGGTCGG |
| *VvUBC15-F* | CGGTTCGTCTCACGGATGT | *VvUBC38-F* | GAAAGGGCAGGAACAAGGATG |
| *VvUBC15-R* | ATCACAGAGCAGGGACTGGA | *VvUBC38-R* | GGCTGTGTATGTGAAGAACCA |
| *VvUBC16-F* | GCTGGGAGATGGCAGAAAAATC | *VvUBC39-F* | GCTCCCAAGGTTCGTTTTCTC |
| *VvUBC16-R* | TGTTCCCTCACTTCCCTCCA | *VvUBC39-R* | AGCAGGGCTCCATTTGTCTT |
| *VvUBC17-F* | CGGCTGGTTTGGTTGATGAG | *VvUBC40-F* | GTCCTTATGCTGGGGGTGTT |
| *VvUBC17-R* | CTGTTTGGGTAGTCGGGTGG | *VvUBC40-R* | CCTTTGTCCTGAATGCCACC |
| *VvUBC19-F* | ACTGTGAAGCAACGTCCTGAA | *VvUBC44-F* | TGCTGATGTTGAAGGCCCAG |
| *VvUBC19-R* | CGTCATGGAACCACCACCTG | *VvUBC44-R* | GGAGGGGAATGAGGGAAGTC |
| *VvUBC20-F* | CCATCTGCTCCCTGTTGACG | *VvUBC45-F* | ACCAAGACAACAACAACAACCC |
| *VvUBC20-R* | TCTCGTACTTGGCCCTGTCT | *VvUBC45-R* | TCCACCATCACCACTCATCA |
| *VvUBC21-F* | CAAGTGAGCGTTGGACACCT | *VvUBC46-F* | GAATACAAAGAGGTGCAGCGGG |
| *VvUBC21-R* | TCCTCACACAACGGCTGAC | *VvUBC46-R* | GCTGGAAAACACCACCCTCA |
| *VvUBC22-F* | GGCTTCAGCTTCAACGAATCA | *VvUBC47-F* | TATGAGGGTGGGGTGTGGAA |
| *VvUBC22-R* | AGAAGCCATCAACGGGGTTT | *VvUBC47-R* | TCCAAGCATACAGAACCTGACAA |
| *VvUBC23-F* | AGCTCCAAAAGTCAAGTGCAAG | *VvUBC51-F* | TCAAGAAGCAGAGTGCGGG |
| *VvUBC23-R* | CGGTTGCGTGAAAAGATGGT | *VvUBC51-R* | AGAGAACACGAATGTACCACCT |
| *VvUBC24-F* | GGAGGGGTTTTCCTTGTTTCTAT | *VvUBC52-F* | CAAGTCACCCCCAGTTCCTC |
| *VvUBC24-R* | AGCAGGGCTCCATTGTTCTTT | *VvUBC52-R* | ATTCGTCTGTCCTTCGCCC |
| *VvUBC25a-F* | CTTGGCAGAGGGAGCATACA | *VvActin-F* | CTTGCATCCCTCAGCACCTT |
| *VvUBC25a-R* | TCCTCGCCTCTTCATTGCC | *VvActin-R* | TCCTGTGGACAATGGATGGA |

**Supplemental Table S2. The organs presented in Fig. S6 from published data GSE36128.**

| **Sample/organ** | **Developmental stages collected** | **Abbreviations** |
| --- | --- | --- |
| Bud | latent bud | Bud - L |
|  | winter bud | Bud - W |
|  | bud swell | Bud - S |
|  | bud burst (green tip) | Bud - B |
|  | bud after-burst (rosette of leaf tips visible) | Bud - AB |
| Inflorescence | young inflorescence (single flower in compact groups) | Inflorescence - Y |
|  | well developed inflorescence (single flower separated) | Inflorescence - WD |
| Flower | flowering begins (10% caps off) | Flower - FB |
|  | flowering (50% caps off) | Flower - F |
| Stamen | pool of stamens from undisclosed flowers at 10% and 50% open flowers | Stamen |
| Pollen | pollen from disclosed flowers at more than 50% open flowers | Pollen |
| Carpel | pool of carpels from undisclosed flowers at 10% and 50% open flowers | Carpel |
| Petal | pool of petals from undisclosed flowers at 10% and 50% open flowers | Petal |
| Tendril | young tendril (pool of tendrils from shoot of 7 leaves) | Tendril - Y |
|  | well developed tendril (pool of tendrils from shoot of 12 leaves) | Tendril - WD |
|  | mature tendril (pool of tendrils at fruit set) | Tendril - FS |
| Leaf | young leaf (pool of leaves from shoot of 5 leaves) | Leaf - Y |
|  | mature leaf (pool of leaves from shoot at fruit set) | Leaf - FS |
|  | senescencing leaf (pool of leaves at the beginning of leaf fall) | Leaf - S |
| Berry Pericarp | fruit set | Berry Pericarp - FS |
|  | post-fruit set | Berry Pericarp - PFS |
|  | véraison | Berry Pericarp - V |
|  | mid-ripening | Berry Pericarp - MR |
|  | ripening | Berry Pericarp - R |
|  | post-harvest withering I (1st month) | Berry Pericarp - PHWI |
|  | post-harvest withering II (2nd month) | Berry Pericarp - PHWII |
|  | post-harvest withering III (3rd month) | Berry Pericarp - PHWIII |
| Berry Skin | post-fruit set | Berry Skin - PFS |
|  | véraison | Berry Skin - V |
|  | mid-ripening | Berry Skin - MR |
|  | ripening | Berry Skin - R |
|  | post-harvest withering I (1st month) | Berry Skin - PHWI |
|  | post-harvest withering II (2nd month) | Berry Skin - PHWII |
| Berry Flesh | post-fruit set | Berry Flesh - PFS |
|  | véraison | Berry Flesh - V |
|  | mid-ripening | Berry Flesh - MR |
|  | ripening | Berry Flesh - R |
|  | post-harvest withering I (1st month) | Berry Flesh - PHWI |
|  | post-harvest withering II (2nd month) | Berry Flesh - PHWII |
| Seed | fruit set | Seed - FS |
|  | post-fruit set | Seed -PFS |
|  | véraison | Seed - V |
|  | mid-ripening | Seed – MR |
| Rachis | fruit set | Rachis-FS |
|  | post-fruit set | Rachis-PFS |
|  | véraison | Rachis-V |
|  | mid-ripening | Rachis-MR |
|  | ripening | Rachis-R |
| Stem | green stem | Stem - G |
|  | woody stem | Stem - W |
| Root | in *vitro* cultivation | Root |
| Seedling | pool of 3 developmental stages | Seedling |
| Total | 54 |  |

**Supplemental Table S3. The water-deficit and salinity stress treatment in published data GSE31677.**

| Treatment | Control | Water-deficit | Salt |
| --- | --- | --- | --- |
| Before treated | Watering and fertilizing regularly | | |
| Two days before experiment | Given exactly 4 l of tap water | | |
| Day 1 | Watered daily with 4 l of water | Watering was completely stopped | 10 mM NaCl and 1 mM CaCl2 |
| Day 2-day 6 |  |  | 20 mM NaCl and 2 mM CaCl2 daily |
| Day 7-day 8 |  |  | 55 mM NaCl and 5.5 mM CaCl2 daily |
| Day 9-day 12 |  |  | 75 mM NaCl and 7.5 mM CaCl2 daily |
| Day 13-day 14 |  |  | 175 mM NaCl and 17.5 mM CaCl_2_ daily |
| Day 15-day 16 |  |  | 250 mM NaCl and 25 mM CaCl_2_ daily |
